# Sex and population mediate the ‘fast growth–short telomere’ association in two species of large herbivores monitored in the wild

**DOI:** 10.1098/rstb.2025.0334

**Published:** 2026-11-12

**Authors:** Florentin Remot, Jean-Michel Gaillard, Benjamin Rey, Emmanuelle Gilot-Fromont, Maryline Pellerin, François Débias, Rebecca Garcia, Corinne Regis, Paul Revelli, Edwige Sautier, Jennifer Dorrens, Hannah Froy, Luise A. Seeker, Sarah Underwood, Rachael V. Wilbourn, Daniel H. Nussey, Jean-François Lemaître

**Affiliations:** ^1^ Institute for Research on Cancer and Aging of Nice (IRCAN), Université Côte d'Azur, INSERM, CNRS, Nice, France; ^2^ Laboratoire de Biométrie et Biologie Évolutive, Université Claude Bernard Lyon 1, Villeurbanne, France; ^3^ Santé Publique Vétérinaire, VetAgro Sup, Marcy l'Etoile, France; ^4^ Service Conservation et gestion durable des espèces exploitées, Direction de la recherche et de l'appui scientifique, Office français de la biodiversité, Chateauvillain, France; ^5^ Institute of Ecology and Evolution, University of Edinburgh, Edinburgh, UK; ^6^ Stanford University School of Engineering, Stanford, CA, USA

**Keywords:** Soay sheep, roe deer, ageing, trade-off, early-life development, telomere

## Abstract

Fast growth is expected to incur physiological costs, notably through accelerated telomere shortening driven by high cell turnover and reactive oxygen species. However, through a systematic literature synthesis, we show that empirical studies exploring this relationship yield mixed results. We investigated the association between early growth and telomere length (TL) in two wild herbivores: the Soay sheep (*Ovis aries*) and the European roe deer (*Capreolus capreolus*). Instead of the expected negative relationship, early-life growth and TL associations were sex- and population-dependent. In Soay sheep, TL correlated negatively with postnatal mass gain in males but not in females. Moreover, the heaviest males at birth appeared buffered against the telomere-shortening associated with rapid postnatal growth. In roe deer, the relationship between reaching a heavy mass at the onset of the first winter and TL was population-specific. Shorter telomeres were only observed in high-quality habitats where fawns reach much heavier body masses, being absent in resource-limited habitats. Additional traits, including secondary sexual characteristics in sheep (e.g. horn type) and male vulnerability to harsh environments in roe deer were further associated with TL. Ultimately, our findings support TL as a relevant somatic marker of the physiological cost of growth, yet underscore its sensitivity to individual attributes and environmental context.

This article is part of the theme issue ‘Developmental circuits in ageing’.

## 1. Introduction

For a long time, natural selection was expected to maximize growth rates, as reaching a heavy adult body mass quickly increases fitness by minimizing both mortality during early life, the life history stage when the risk of death is particularly pronounced [1], and age at first reproduction. However, empirical evidence that animals often grow below their physiological maximum has led to the hypothesis that fast growth may incur long-term fitness costs [2,3]. This negative association between early growth and late-life survival is one cornerstone of life history theory [4] and a key prediction of evolutionary theories of ageing (reviewed in [5]). For instance, substantial allocation of resources towards growth might compromise somatic maintenance, leading to the progressive accumulation of damage underpinning the ageing process (disposable soma theory, [6,7]). Alternatively, while natural selection finely tunes gene expression to optimize growth early in life, this optimization may deteriorate later in life, as age-related shifts in optimal expression occur and selection becomes too weak in late life (i.e. the so-called selection shadow [8]) to restore gene expression to those optimal levels (developmental theory of ageing, [9–11]). In line with these predictions, the detrimental effect of fast growth on ageing (e.g. reduced survival probabilities in late life) has been documented in both laboratory models [12–15] and populations of animals in the wild [16–18]. Among the molecular mechanisms that could mediate the relationship between growth and ageing, telomere dynamics has attracted much attention [19–23] and appears compatible with both disposable soma and developmental theories of ageing [5].

Telomeres, the protective caps of the chromosome end, are composed of repeated nucleotide sequences (TTAGGG in most vertebrates) located at the extremity of chromosomes [24]. They prevent the erroneous activation of DNA repair pathways at the ends of linear chromosomes [25], play a key role in chromosomal segregation during cell division and inhibit chromosomal fusions during mitosis [26]. Telomeres shorten during each round of cell replication owing to the ‘end-replication problem’ and are highly sensitive to oxidative damage owing to their guanine-rich composition [27–29]. When they reach a critical threshold, the shortest telomeres in a given cell trigger a DNA damage response, causing cell cycle arrest and ultimately apoptosis [30]. Although telomerase can partially counteract this shortening by synthesizing new telomeric repeats [31], it elongates only a subset of telomeres during each cell cycle, so that telomere attrition still occurs in telomerase-expressing somatic cells [32]. In addition, while some species maintain telomerase activity throughout life, others, such as most mammals, show repression of somatic telomerase shortly after birth [33]. As a consequence, telomeres generally shorten with increasing age in most vertebrates studied to date, although the rate of decline varies across vertebrate classes [34].

Two (non-mutually exclusive) mechanisms have been proposed to link rapid growth and accelerated telomere erosion: increased rate of cell division and altered oxidative balance [20]. On one hand, growth occurs through increases in cell size and/or number [1]. Because the allometric scaling of cell size is insufficient to account for the large difference in body size observed across the tree of life [35], reaching a large body size necessarily requires more cell divisions. Thus, because telomeres shorten with each cell division [28], elevated growth rates are expected to accelerate telomere shortening by increasing the cumulative number of replicative cycles. The second mechanism proposes that increased investment in growth elevates energetic demand at the expense of cellular protective processes (e.g. antioxidant defences, repair or renewal of oxidized molecules), promoting the accumulation of reactive molecules and exacerbating telomere damage (see [36] for a meta-analysis).

Whatever the mechanisms at play, a growing body of evidence indicates that most telomere loss in endotherms occurs during early life, particularly during the growth period in both mammals [37–39] and birds [40,41] (but see [34] for some counter-examples). By contrast, ectotherms typically exhibit indeterminate growth, which involves fundamentally different growth patterns and telomere dynamics that limit direct life-stage comparisons [42]. However, in endotherms, the growth–telomere relationship remains poorly explored in wild mammals compared with avian species. This gap is particularly problematic because mammals exhibit growth trajectories that differ markedly from those of birds, typically reaching asymptotic body size well after sexual maturity, following a comparatively slower growth rate [20]. In mammals, early life, particularly the pre-weaning period, represents a critical developmental window characterized by rapid somatic growth and strong dependence on both maternal allocation and environmental resource availability [43]. Although ecological constraints are expected to intensify resource allocation trade-offs between growth and somatic maintenance [4,44], how environmental harshness shapes the growth–telomere relationship in natural mammalian populations remains largely unknown. Furthermore, this early developmental stage is also often characterized by sex-specific growth patterns. In many polygynous mammals, males either exhibit faster early mass gain or begin allocating substantial resources to the development of secondary sexual traits (e.g. horns or antlers in artiodactyls) shortly after birth [45]. Such differential allocation may result in higher physiological costs in males, potentially accelerating telomere attrition during early life compared with females. Evaluating how environmental conditions, early-life growth dynamics and sex interact to shape telomere dynamics during this critical early-life stage is therefore a crucial step to better understand mammalian life histories.

While the foundational synthesis by Monaghan & Ozanne [20] established a critical framework for understanding the relationship between growth and telomere dynamics, the literature on this topic has expanded rapidly over the past eight years. Given this continuous influx of new empirical data, an updated synthesis is now required to evaluate the current consensus. Therefore, the present study pursued two main objectives. First, we conducted an updated systematic literature synthesis to quantify the current state of evidence regarding the growth–telomere relationship in non-model vertebrates. Second, to address the well known scarcity of mammalian data in this specific field, we empirically investigated the relationship between early-life growth and telomere length (TL) in two distinct wild ungulate species with contrasting life history strategies: the Soay sheep (*Ovis aries*) and the roe deer (*Capreolus capreolus*).

We tested the hypothesis that rapid early growth is associated with a higher rate of telomere shortening in three populations of mammals intensively monitored in the wild. We took advantage of detailed early-life growth and TL data collected from two large herbivore species that are subject to long-term, individual-based monitoring. In the Soay sheep population on the remote St Kilda archipelago, we tested relationships between TL at four months of age and body mass at birth, body mass at four months of age and horn growth over the first four months of life. In two French populations of roe deer experiencing contrasting environmental conditions, we investigated the relationships between adult TL and body mass (measured within the first 20 days of life for one population, and after the weaning period at approximately eight months of age for both populations). In this species, we expected environmental harshness to have contrasting effects: resource limitation might negatively impact adult TL directly, but paradoxically limit early-life growth and its deleterious consequences on telomere dynamics.

## 2. Material and methods

### (a) Systematic literature search

To provide an updated quantitative synthesis of the relationship between early-life growth and telomere dynamics, we conducted a systematic literature search designed to expand the foundational dataset originally compiled by Monaghan & Ozanne [20]. We systematically searched the Web of Science Core Collection database using the search terms ‘*telomere*’*AND* ‘*growth*’. To ensure the retrieved literature was relevant to our ecological and evolutionary framework, we restricted our search to the Web of Science categories of *Multidisciplinary Sciences, Ecology* and *Evolutionary Biology*. The search was conducted to include all literature published up until January 2026. Following the initial retrieval, studies were screened based on the criteria established by Monaghan & Ozanne [20], focusing on empirical studies that explicitly tested the association between somatic growth rates (or body mass changes) and TL or attrition in vertebrates. However, to specifically address ecological and evolutionary dynamics in natural systems, we decided to strictly exclude studies focusing on humans, traditional laboratory model organisms and artificially selected lines. Our screening was therefore restricted exclusively to non-model species, encompassing wild, semi-captive or wild-derived populations (as already done in previous meta-analysis [34]). Through this updated search and filtering process, we identified and extracted outcomes from 32 newly published studies. These were added to the 14 relevant studies initially compiled in the 2018 review (after applying our exclusion criteria to their dataset), yielding a final updated dataset of 46 studies (detailed in supplementary material, table S1).

### (b) Study species and populations

The Soay sheep is a primitive sheep breed that has inhabited the St Kilda archipelago (Scotland, UK 57°49′ N, 8°34′ W) for at least the last two millennia. Individuals in the Village Bay population on Hirta Island have been the subject of an intensive individual-based study since 1985 [46]. Soay sheep grow and develop rapidly over the first year of life. Sheep are a highly polygynous species with pronounced sexual size dimorphism and male-biased mortality rates [46]. Although the sexes are similar in size at birth, their growth rates diverge rapidly, with adult males being much heavier and larger than females [46]. The sheep show genetically controlled variation in horn morphology. Normal-horned individuals grow horns throughout life, although these are much smaller in females and are an important secondary sexual trait in males [47]. Scurred and polled individuals grow barely visible horns [48].

For the present study, we focused on growth across the first four months of life in both sexes. In April and May, females give birth to singleton or twin lambs. Around 20% of births result in twins, although this varies annually. Field teams capture lambs within the first few weeks after birth, and lambs are weighed and uniquely marked with ear tags. Most lambs are caught within their first week of life. Each summer (August), a larger field team visits St Kilda for around two weeks to capture and monitor individuals. We set up a series of corral traps using nets across our study area and typically captured around 60% of the resident population (200–300 sheep). At capture, all sheep are weighed and measures of skeletal size, horn length and circumference are taken. Blood samples are also collected, and blood is separated into plasma and buffy coat (crude white blood cell) fractions and stored at –20°C [38]. Lambs captured in their first summer of life are around four months old. They are weaned and are becoming independent of their mothers. We used birth mass and summer mass at four months of age (called juvenile mass hereafter) and TL data derived from summer blood samples in our analyses (see table 1A).

The roe deer data stem from two enclosed forest populations managed by the Office Français de la Biodiversité (OFB). These two populations have been intensively monitored using a long-term capture–mark–recapture programme since 1975 and 1977 for Trois-Fontaines and Chizé, respectively. Trois-Fontaines and Chizé experience highly contrasting environmental conditions. Trois-Fontaines (northeast France, 48°43′ N, 4°55′ E) is characterized by a high-quality forest environment with rich soil and high food availability. By contrast, Chizé (west France, 46°050′ N, 0°250′ W) is a poor-quality environment with a low-productivity forest and frequent summer droughts [49], which likely explain the lower winter juvenile survival [50], as well as the lower adult body mass of roe deer in Chizé compared with Trois-Fontaines [16].

Each year between April and May, newborn fawns have been systematically searched for, captured and marked using ear tags since 1985 in both populations. The age of fawns is estimated from behaviour and physical traits [51], and only fawns aged between birth and 20 days of life are considered as known-age fawns. The average age of fawns at marking is 4 days. In both populations, 12 days of capture are organized each year between December and March [50]. Once an individual is captured, a basic clinical examination is performed, its sex and body mass (to the nearest 50 g) are recorded, and a blood sample is collected (1 ml per kg of body mass, with a maximum of 20 ml) into heparin–lithium collection tubes (4 ml Vacutainer, BD Medical) from the jugular vein. Blood samples were processed immediately to separate the buffy coat fraction and stored at –80°C. Individuals are equipped with individually recognizable ear tags and collars (either numbered, VHF or GPS collars), given a unique ID, and closely monitored throughout their lifetime using subsequent winter captures and field observations. All individuals included in our analyses were of known age because they were caught either as newborns in spring (see [52] for further details) or at *ca* eight months old during winter captures, when they still have their milk teeth (most often incisors and always a distinctive tricuspid third premolar [53]). In addition, our dataset was strictly filtered to include only individuals that were successfully captured and weighed at eight months old. Furthermore, for 46 individuals within the Trois-Fontaines population, we obtained an early-life mass measurement from fawns captured between birth and 20 days of age. Overall, in our roe deer analyses, we used an early body development index calculated as the deviation between the observed body mass of a given fawn at age *x* and the expected average mass of a fawn at age *x* based on the generalized additive model (GAM) relationship between body mass and age across all fawns marked during the study (about 1500 individuals only in Trois-Fontaines). We also used winter fawn mass corrected for the date of capture, as roe deer fawns still grow throughout the winter (in both populations), and TL data derived from adult blood samples (see below and table 1B).

**Table 1.** List of traits included in the analyses of relative telomere length (TL) in Soay sheep (A) and roe deer (B). CH, Chizé; TF, Trois-Fontaines.

(A) Soay sheep trait	distribution, units and sample size	predictions on TL
age at sampling (in summer)	from 93 to 137 (in days), (841)	shorter telomeres in older individuals
standardized birth mass	from 0.92 to 4.62 (in kg), (841)	heavier individuals at birth have shorter telomeres (i.e. cost of prenatal growth)
juvenile mass	from 5.4 to 19.95 (in kg), (841)	for a given birth body mass, heavier individuals in August have shorter telomeres (i.e. cost of early-life growth)
sex	male (387), female (454)	males are usually bigger than females, and should have shorter telomeres
horn type	normal (480), not normal (361)	normal-horned individuals should have shorter telomeres owing to the cost of horn growth
twin status	lambs without twin (676), with a twin (165)	twins are smaller and rarely survive their first year; they should have shorter telomeres
**(B) roe deer trait**	**distribution, units and sample size**	**predictions on TL**
age at sampling (in winter)	from 1 to 14 (in years), (250) (CH and TF)	shorter telomeres in older individuals, with a stronger effect in CH
early development index (standardized body mass at 4 days of age)	from 1.49 to 3.05 (in kg) (62) (TF)	heavier individuals have shorter telomeres (i.e. cost of prenatal growth)
standardized fawn mass (at eight months of age)	from 8.24 to 21.03 (in kg), (137) (CH); from 10.24 to 20.95 (in kg) (113) (TF)	heavier fawns have shorter telomeres (cost of prenatal and early-life growth)
mass at sampling	from 8.2 to 28.15 (in kg), (137) (CH); from 10.5 to 27.9 (in kg), (113) (TF)	heavier individuals have shorter telomeres (cost of growth)
sex	male (61), female (76) (CH); male (57), female (56) (TF)	no sex effect owing to similar birth mass and growth rates between males and females

### (c) Telomere length measurement

For both species, TL analyses were conducted between September 2017 and spring 2018. In Soay sheep, relative telomere length (RTL) was measured from blood samples collected between 1998 and 2016 in four-month-old individuals (841 individuals). These samples had been stored as buffy coats at –20°C for a period ranging from 2 to 20 years prior to analysis. In roe deer populations, RTL was measured from blood samples collected during the 2016 and 2017 winter campaigns in both eight-month-old and adult individuals (113 and 137 measurements encompassing 94 and 120 individuals for Trois-Fontaines and Chizé, respectively). These buffy coat samples were initially stored at –80°C for approximately three months before DNA extraction, after which the extracted DNA was stored at –80°C for an additional 3 to 18 months before telomere measurement. For both Soay sheep and roe deer, RTL was estimated at the Institute of Ecology & Evolution (Edinburgh) following a similar method (previously described in [38,54,55]). In brief, genomic DNA for each sample was extracted from the buffy coat layer (white blood cells) and eluted to 150 µl in the elution buffer [38]. Relative leukocyte TL was measured using real-time quantitative polymerase chain reaction (qPCR). Following previously established methods in sheep and cattle [56,57], we used beta-2-microglobulin as our non-variable copy number reference gene. On the same qPCR plate, the reference gene and telomere samples were run in different wells. To account for plate variance, each plate also contained eight calibrator samples and two non-template controls made using nuclease-free water. Serially diluted samples of the calibrator were used to estimate a standard curve, and all samples (controls, calibrators and tests) were conducted in triplicate. The amplification curves were adjusted for baseline fluorescence using the *linregpcr* software program (version 2016.0, [58]), and well-specific reaction efficiencies and cycle quantification (Cq) values were computed. Samples were discarded if the coefficient of variation across triplicate Cq values and triplicate PCR efficiency values for either amplicon was greater than 5%, or if the efficiency of at least one of their triplicate reactions differed by more than 5% from the mean efficiency across all wells on that plate for the relevant amplicon. RTL was then computed for each sample (as per [59]) using average reaction efficiencies for each plate and Cq computed by *linregpcr* as follows:

$$\begin{array}{r} RTL\;=\;\frac{Etel^{\left(Cqtel\left[Calibrator\right]\;-\;Cqtel\left[Sample\right]\right)}}{Eb2m^{\left(Cqb2m\left[Calibrator\right]\;-\;Cqb2m\left[Sample\right]\right)}}, \end{array}$$

where Etel and Eb2m are the mean reaction efficiencies for the respective amplicon group across all samples on a given plate, Cqtel[Calibrator] and Cqb2m[Calibrator] are the average Cq values for the relevant amplicon across all calibrator samples on the plate, and Cqtel[Sample] and Cqb2m[Sample] are the average of the triplicate Cq values for the sample for each amplicon [38].

### (d) Statistical analysis

Statistical analyses were performed separately for Soay sheep and roe deer using R version 4.5.0 [60]. Data visualization was performed using *ggplot2* version 3.5.2 [61]. Prior to modelling, we checked for collinearity among continuous predictors to ensure that highly correlated variables (Pearson's *r* > 0.7) were not included in the same models [62].

#### (i) Soay sheep

To test the relationship between TL and growth in lambs (*n* = 841 individuals; 387 males and 454 females), we ran linear mixed models with RTL as a response variable. We included fixed effects of standardized birth mass, juvenile mass (at four months of age in the Soay sheep), age in August in days, the sex of individuals, horn type (normal or not normal, i.e. scurred and polled horns), and twin status (whether a given lamb has a twin or not), since twins are lighter than singletons both at birth and in August [63] (table 1A). To account for variation in capture timing, birth mass was standardized to the median age of lamb capture (2 days old). Specifically, we fitted a linear model with capture mass as the response variable and age at capture (in days) as the explanatory variable, and we extracted the predicted mass at 2 days of age for each individual. Crucially, by including both birth mass and juvenile mass in the same additive model, we statistically controlled for initial size. Thus, the effect of juvenile mass in our models specifically captures the variance associated with postnatal mass gain, rather than consistent among-individual differences in overall body size. We also included biologically meaningful interactions to investigate sex-specific effects of growth on telomere dynamics. Specifically, we included the interaction between sex and birth mass, the interaction between sex and juvenile mass to test whether postnatal growth could be different between males and females, as well as the interaction between sex and horn types to test whether the cost of growing normal horns could be different between the sexes. To account for variation associated with measurement error of RTL, we included the qPCR plate and row as random factors in all models (following [38]). We also included the year of sampling and the identity of the mother as random effects in all models. All models were run using the package *glmmTMB* version 1.1.13 [64]. To interpret and visualize our results, we used the *emmeans* package version 1.75 [65]. Specifically, we calculated the estimated marginal means and 95% CIs to plot pairwise contrasts for our categorical predictors (i.e. sex, horn type and twin status). Additionally, to visualize statistically significant interactions, involving continuous variables (e.g. sex-specific growth dynamics), we extracted the estimated marginal trends (slopes) for each group using the *emtrends* function.

#### (ii) Roe deer

We tested the effect of early-life body mass metrics on RTL measured at the time of sampling (which included both eight-month-old fawns and adults) using linear mixed models. As a previous study revealed that the relationship between RTL and age differed between populations (see [56]), we tested for such environmental differences by including the population as an interacting term with age. Winter fawn mass (at *ca* eight months old) was corrected by the Julian date of capture, considering population-specific growth rate [16]. We used the same modelling procedure as for the Soay sheep to evaluate the relationship between RTL and several predictors. The full model included RTL as the response variable. Because individuals were sampled for RTL at various stages of their life (ranging from eight months to 14 years of age), we included their exact age at capture (in years), fitted as a continuous variable to control for baseline age-related telomere attrition. The model also included body mass at capture, standardized fawn mass (at eight months of age), sex and population as fixed effects (table 1B). To account for population differences in telomere attrition with age, we included the interaction between population and age at capture. Crucially, to test whether environmental harshness modulates the physiological cost of early growth, we included the interaction between population and fawn mass. To account for the non-independence of data resulting from longitudinal monitoring, individual identity was included as a random effect in the model, as well as the cohort. To account for variation associated with measurement error of RTL, we included the qPCR plate as a random factor in all models.

Finally, we investigated the effects of the early body development index on RTL in a separate analysis, as this trait was only available for individuals marked within their first 20 days of life in Trois-Fontaines (62 RTL measurements across 46 individuals). We first regressed body mass on the exact age at capture across all fawns marked and weighed within their first 20 days of life. To do so, following Plard *et al.* [66], we fitted a GAM of fawn body mass against age in days, extracted individual residuals and added them to the GAM-predicted population mean mass at the median capture age (4 days). As roe deer fawns were marked over a much longer early-life period than sheep, we cannot assume a linear growth pattern to measure early-life development as we did for sheep. The effect of this standardized early body development index on RTL was tested in a separate model specifically dedicated to this population, using the same baseline covariates.

## 3. Results

### (a) Systematic literature synthesis of the growth–telomere relationship

Our updated systematic search identified 46 studies meeting our inclusion criteria, from which we extracted 66 distinct relationships between body growth and telomere dynamics (figure 1; supplementary material, table S1). Taxonomically, the available literature on wild and non-model vertebrates is heavily skewed: the vast majority of studies have been conducted on avian species (*n* = 34, 74%), whereas mammals have received considerably less attention, accounting for only 4% (*n* = 2) of the dataset. The remaining 22% of the dataset consists of studies conducted on other vertebrate taxa (4, 5 and 1 studies for reptiles, fish and amphibians, respectively). Across the 66 extracted relationships, we observed a striking lack of consensus regarding both the presence and the direction of the growth–telomere association. Exactly half of the reported relationships were not statistically significant (*n* = 33), while negative (*n* = 22) and positive (*n* = 11) associations between body growth and TL accounted for one-third and one-sixth of the case studies, respectively. When breaking down the literature by study design, this mixed pattern persisted. Among the 18 relationships extracted from the 15 strictly experimental studies that successfully manipulated growth rates, seven showed a negative association, nine were not statistically significant and two were positive. Similarly, among the 48 relationships extracted from correlative studies (including those where experimental treatments failed to successfully manipulate growth trajectories), half of the results were not statistically significant (*n* = 24, 50%), whereas the remaining relationships were divided between negative (*n* = 15, 31%) and positive (*n* = 9, 19%) associations.

**Figure 1.**
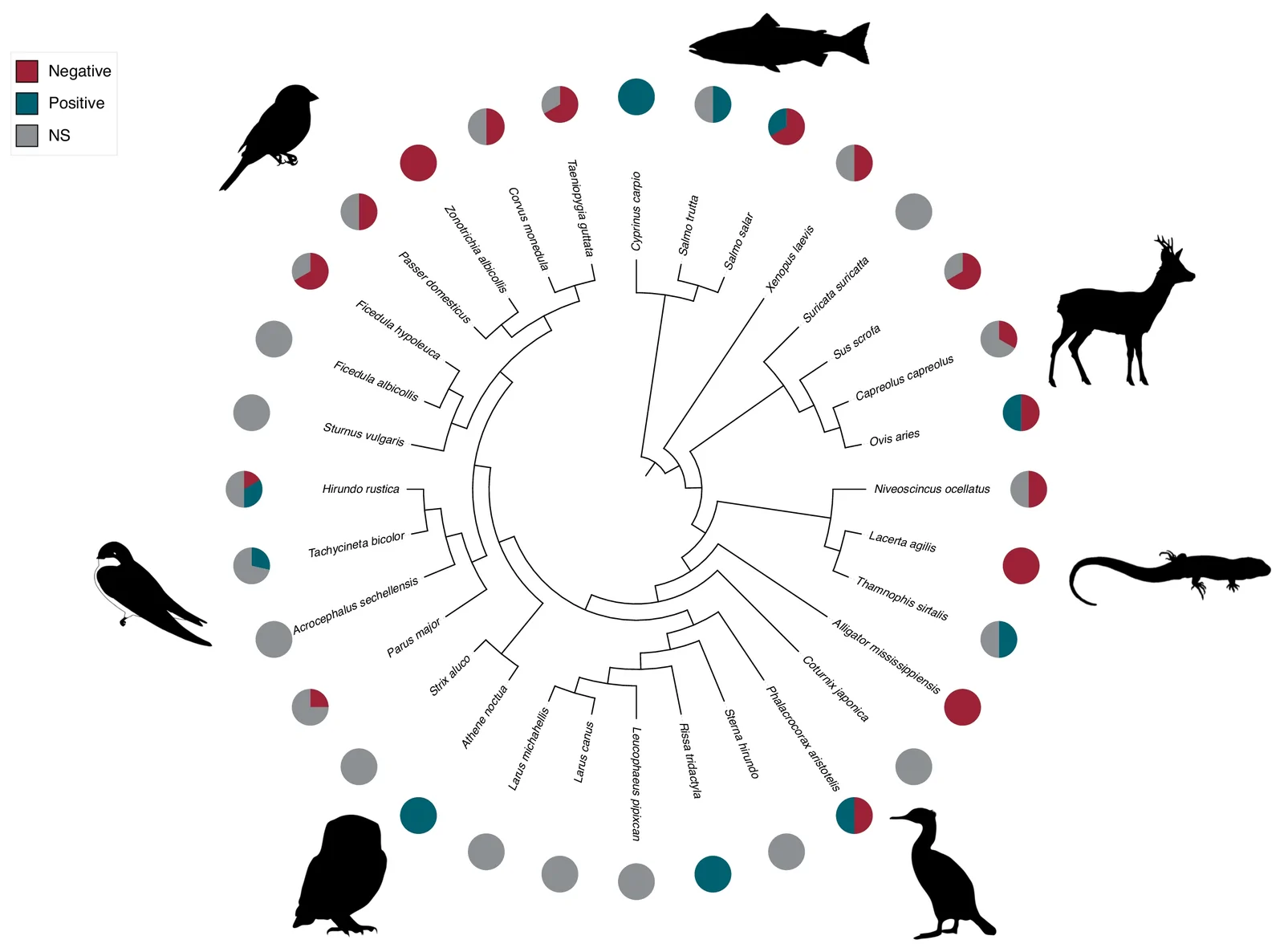
Phylogenetic distribution of the observed relationships between early growth and telomere length (TL) in vertebrates in the wild. The phylogenetic tree summarizes 46 studies examining the association between early-life mass or size and TL in wild and semi-captive non-model species. Pie charts at the branch tips represent the proportion of reported effects for each species: negative (red), positive (blue) and not statistically significant (NS) associations (grey). Note that data for Soay sheep (*Ovis aries*) and roe deer (*Capreolus capreolus*) incorporate the findings of the present study. The complete review table is available in supplementary material, table S1 .

### (b) Soay sheep

To identify the factors associated with RTL in Soay lambs, we ran a global model including birth mass, juvenile mass, horn phenotype and their respective interactions with sex as fixed effects. The results from this global model revealed that telomere dynamics in relation to early growth were sex-specific (table 2).

**Table 2.** Summary of the global model testing the predictors of relative telomere length (RTL) in Soay lambs. Estimates, s.e. and *p*-values are extracted from the global model assessing the associations between RTL and the traits described in table 1A. Reference levels are ‘female’ (sex), ‘normal horn’ (horn type) and ‘singleton’ (twin status). Statistically significant effects are in bold.

trait	estimate	s.e.	*p*-value
sex	0.0008	0.094	0.993
juvenile mass	–0.003	0.008	0.699
**horn type**	**0.080**	**0.026**	**0.002**
twin status	–0.039	0.026	0.138
standardized birth mass	–0.052	0.032	0.107
age at sampling	0.0001	0.001	0.966
**sex × juvenile mass**	**–0.019**	**0.009**	**0.049**
**sex × standardized birth mass**	**0.140**	**0.044**	**0.001**
sex × horn type	–0.056	0.050	0.265

Specifically, we found a detectable interaction between sex and birth mass effects (estimate = 0.140 ± 0.044 s.e.). In male lambs, heavier individuals at birth had longer telomeres (slope = 0.088 ± 0.034 s.e.), but the association was not found in females (slope = –0.052 ± 0.032 s.e.) (table 2; figure 2A). The influence of early postnatal growth on RTL, assessed as the effect of juvenile mass at four months of age controlling for standardized birth mass effect, differed between sexes (estimate = –0.018 ± 0.009 s.e.). Higher postnatal mass gain was associated with shorter RTL in males (slope = –0.022 ± 0.007 s.e.) but not in females (slope = –0.003 ± 0.008 s.e.) (table 2; figure 2B).

**Figure 2.**
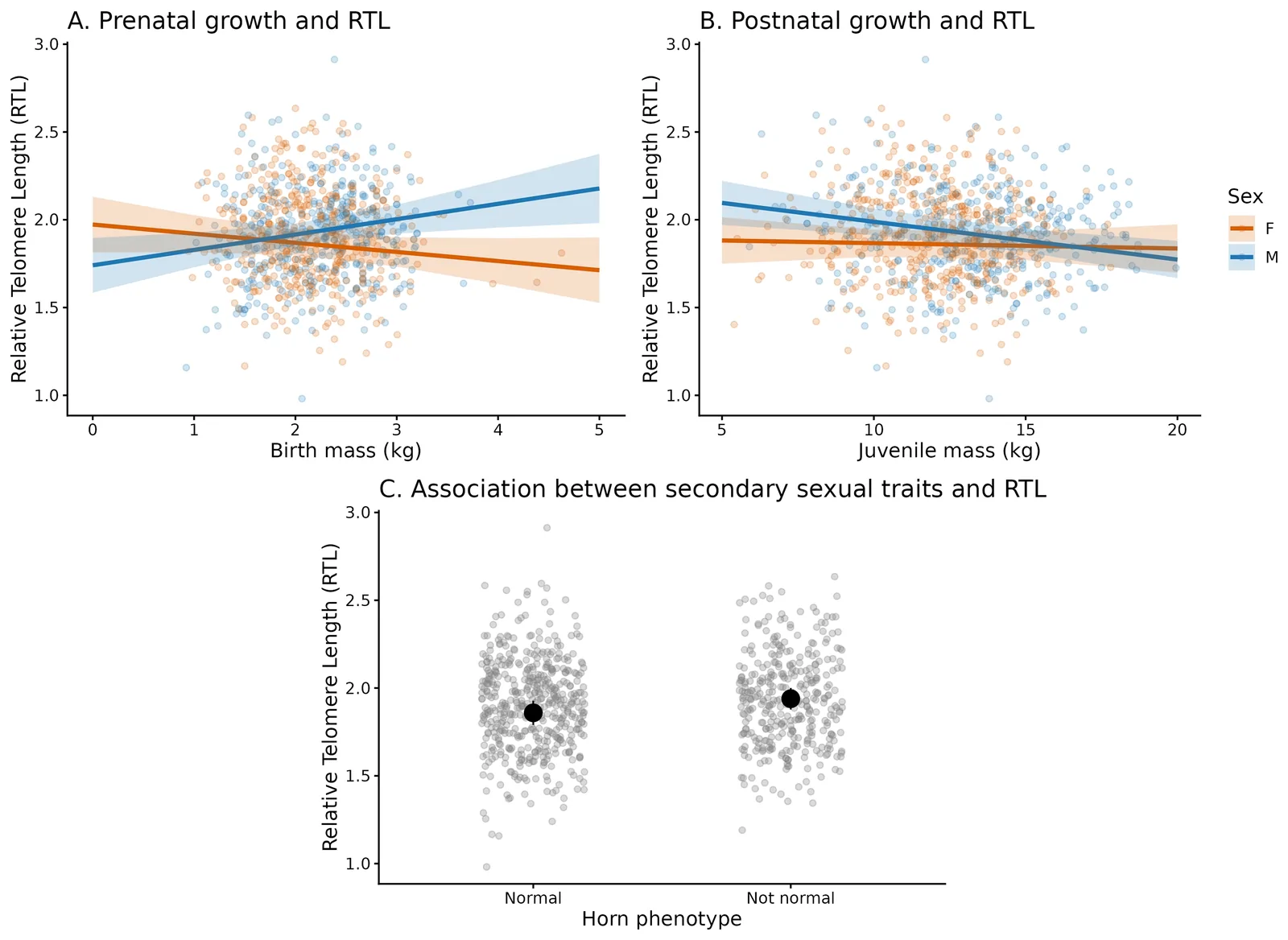
Sex-specific effects of early-life growth and secondary sexual traits on relative telomere length (RTL) in the Soay sheep. (A) Scatterplot representing the interactive effect of birth mass and sex on RTL. (B) Scatterplot representing the interactive effect of postnatal growth (juvenile mass coryrected for birth mass) and sex on RTL. For (A) and (B), solid regression lines and shaded areas represent the marginal predicted means and 95% confidence intervals extracted from the global model. Predictions were computed by holding all other fixed effects present in the global model constant at their mean values or reference levels. (C) Jitter plot illustrating the association between secondary sexual traits and RTL, showing the distribution of RTL across horn phenotypes (Normal versus Not normal). Semi-transparent points represent raw data; large markers and associated vertical lines represent the group means and s.e.

Finally, RTL was influenced by horn type, with individuals growing normal horns exhibiting shorter telomeres than those with other horn types (estimate = –0.052 ± 0.0252 s.e.) (table 2; figure 2C).

### (c) Roe deer

Telomere dynamics in roe deer markedly differed between populations. To investigate these dynamics, our global model included age, standardized fawn mass at the onset of winter, sex and their respective interactions with the population of origin. The results from this full model revealed that both age and standardized fawn mass exhibited different relationships with RTL between the two contrasting populations (table 3).

**Table 3.** Summary of the global model testing the predictors of relative telomere length (RTL) in roe deer. Estimates, s.e. and *p*-values are extracted from the global model assessing the associations between RTL and the traits described in table 1B. Reference levels are ‘Chizé’ (Population) and ‘female’ (Sex). Statistically significant effects are in bold.

trait	estimate	s.e.	*p*-value
age at sampling	–0.008	0.006	0.154
**population**	**0.379**	**0.184**	**0.039**
mass at sampling	0.001	0.004	0.862
standardized fawn mass at eight months of age	0.007	0.011	0.492
**age × population**	**0.021**	**0.008**	**0.007**
**population × standardized fawn mass at eight months of age**	**–0.028**	**0.012**	**0.022**
population × sex	0.111	0.062	0.073
standardized fawn mass at eight months of age × sex	–0.001	0.012	0.949

The influence of age on RTL differed between populations (interaction term estimate = 0.021 ± 0.008 s.e.). RTL tended to increase with age in Trois-Fontaines (slope = 0.012 ± 0.007 s.e.), whereas it did not in Chizé (slope = –0.009 ± 0.006 s.e.) (table 3; figure 3A). Likewise, the effect of standardized fawn mass at eight months of age differed between populations (interaction term estimate = –0.028 ± 0.012 s.e.). Heavier fawns had shorter RTL in the high-quality environment of Trois-Fontaines (slope = –0.021 ± 0.01 s.e.), but not in the resource-limited environment of Chizé (slope = 0.007 ± 0.009 s.e.) (table 3; figure 3B). The sex-by-population interaction was not statistically significant (*p* = 0.073), though the data suggested that males tend to display shorter RTL than females in Chizé but not in Trois-Fontaines (table 3; figure 3C).

Lastly, focusing on the fawns marked within their first 20 days of age at Trois-Fontaines (i.e. with known birth dates), we ran an additional global model, including the early development index. The results indicated that this trait was not associated with RTL measured later in life (supplementary material, table S2).

**Figure 3.**
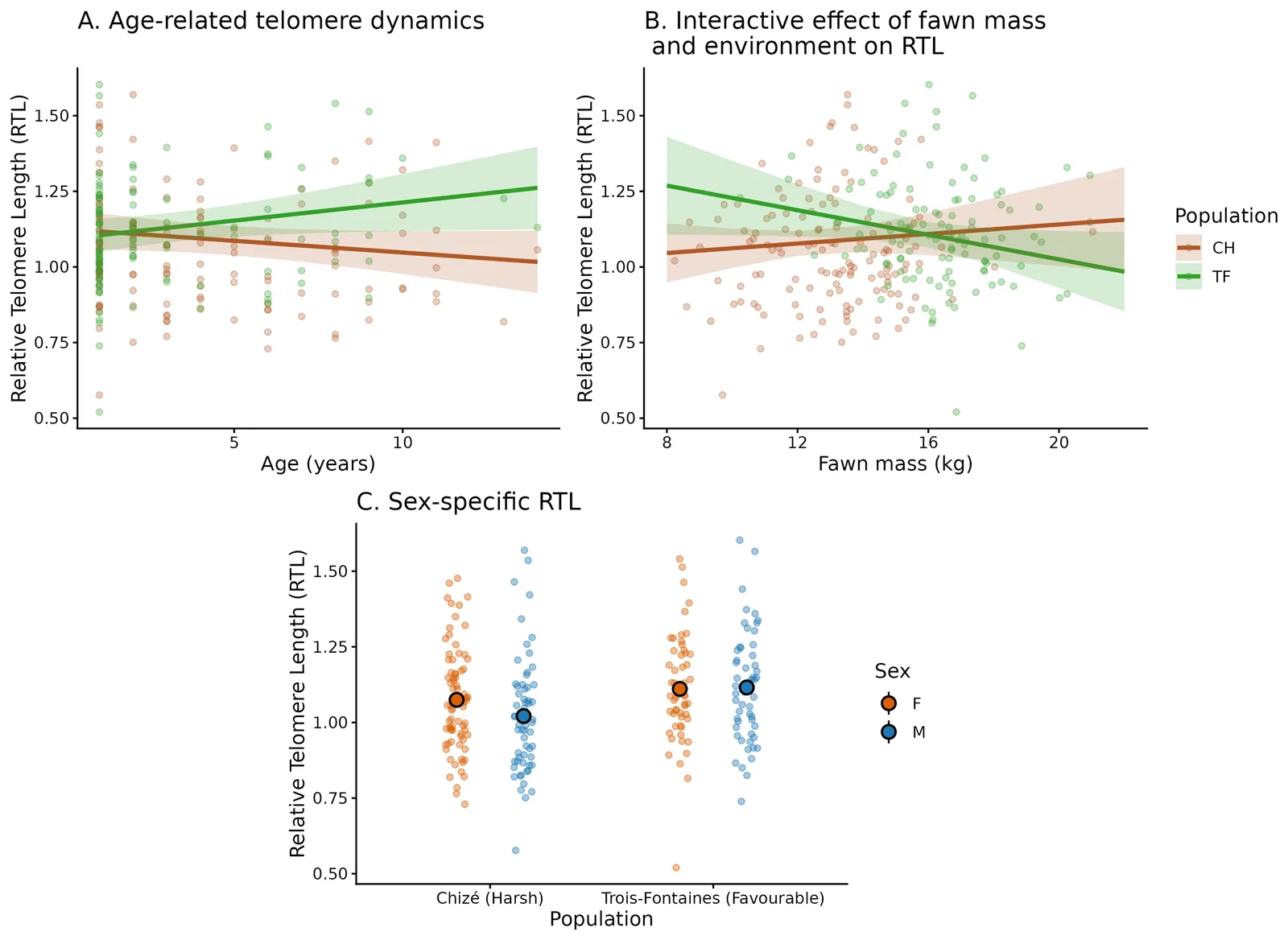
Environmentally modulated effects of age, mass at eight months of age, and sex on relative telomere length (RTL) in the roe deer. (A) Scatterplot illustrating the interactive effect of age and population on RTL. (B) Scatterplot representing the interactive effect of fawn mass at eight months of age and population on RTL. For (A) and (B), solid regression lines and shaded areas represent the marginal predicted means and 95% confidence intervals extracted from the global model. Predictions were computed by holding all other fixed effects present in the global model constant at their mean values or reference levels. (C) Jitter plot illustrating sex-specific RTL per population. Semi-transparent points represent raw data; large markers and associated vertical lines represent the group means and standard errors (s.e.). CH, Chizé; TF, Trois-Fontaines.

## 4. Discussion

While the observed responses of RTL to early body growth align with expected physiological costs, they crucially highlight that this relationship is highly both population- and sex-specific. The association between rapid juvenile growth and short RTL became apparent when accounting for sex-specific growth in Soay sheep and population effects in roe deer. Importantly, our correlative approach prevents us from revealing causal relationships between early growth and telomere dynamics because potentially confounding effects of individual heterogeneity and variation in environmental quality across years were not controlled for. Nevertheless, documenting such population- and sex-specific effects in the wild remains highly relevant. This is especially true given the specificities of mammalian telomere biology, which make mammals highly vulnerable to telomere attrition in early life. Indeed, unlike many avian and ectothermic species that maintain telomerase activity in somatic tissues [42,67], most large mammals repress telomerase expression shortly after embryonic development. This repression is thought to have evolved primarily as an anti-tumour mechanism rather than as a consequence of resource limitation [33,68]. Consequently, large mammals may be especially sensitive to the replicative toll of rapid early growth, as telomere attrition incurred during early development is unlikely to be rescued later in life. For instance, the strength of natural selection might be too weak in late life to restore telomerase expression, when the benefits of protecting DNA sequences and thus maintaining genomic stability could outweigh the benefits of limiting tumour proliferation [5]. In line with our results, our literature review of 46 studies examining the relationship between growth and telomere dynamics in vertebrates in the wild or in semi-captive conditions highlights striking inconsistencies, with the expected negative association being frequently absent (figure 1; supplementary material, table S1). Furthermore, mammals were under-represented in our survey. Besides the fact that studies on ectothermic vertebrates outnumbered those on mammals, the empirical landscape we retrieved was heavily skewed towards avian models, leaving large gaps in our understanding of mammalian somatic maintenance. Our findings from intensively monitored populations of two species of large herbivores in the wild provide a potential explanation for the heterogeneous outcomes reported from our literature survey. Specifically, our models suggest that the association between early-life growth and telomere dynamics is highly variable and can differ between sexes (as observed in Soay sheep) or population-specific traits (as observed in roe deer). This could explain why previous studies that mostly focused on only one sex or one population may have failed to detect such effects, thereby contributing to the high proportion of null results observed in the literature.

In Soay sheep, the telomere shortening associated with early postnatal growth was sex-specific, with rapid growth being negatively associated with RTL in males but not in females. Previous work in this population revealed a sex difference in TL, with males having shorter telomeres than females from 3 years of age onwards [55]. Our results build upon these findings by suggesting that the sex-specific somatic trade-offs leading to these later-life differences could already be set in motion during the first months of life. This association is in line with the highly dimorphic life history of this species. As Soay rams intensively compete for mating, there is strong selection on adult male size. To reach a competitive physical stature, young males must undergo periods of accelerated and sustained somatic growth compared with females [69]. In support, males gained on average 1.16 ± 0.126 (s.e.) kg more than females within their four months of life. At the cellular level, fast early growth requires higher rates of cell proliferation and elevated metabolic activity, while diverting resources from antioxidant and repair machinery, which can in turn elevate oxidative stress [70,71]. Since oxidizing molecules are primary drivers of telomere attrition [20], this provides a potential biological pathway for the specific telomere erosion associated with fast growth in male lambs. Interestingly, this postnatal trade-off was coupled with a sex-specific dependency on prenatal allocation. In Soay sheep, male fetuses generally require a higher maternal allocation, resulting in a slightly heavier birth mass compared with females (by 60–80 g [69], in line with the 59 g difference observed in our dataset), which in turn is positively associated with longer telomeres. This suggests that male lambs receiving a substantial initial maternal allocation are better equipped to buffer the cellular trade-offs of their subsequent demanding postnatal growth phase. By contrast, females, with a smaller growth rate, do not exhibit such patterns of telomere attrition during early development, nor do they rely as heavily on initial mass to preserve their somatic integrity. Such differential investment might be driven by sex-specific placental functions, which can mediate sexually dimorphic nutrient and antioxidant transfer from the mother [72]. These patterns highlight that the developmental window during which a trade-off between growth and TL can occur depends on species-specific life histories. For instance, in wild boars (*Sus scrofa*), which produce large litters, the somatic burden appears to occur earlier, during the embryonic stage [73]. By contrast, for precocial species like Soay sheep [74], the physiological toll is shifted later in development, particularly during the critical postnatal period. While our approach does not allow us to pinpoint the exact timing of telomere shortening, our results suggest that the critical early growth phase required to reach a threshold mass for overwinter survival [46,69] is ultimately associated with shorter telomeres by the end of the summer.

Beyond individual attributes, such as sex, our comparative approach reveals that environmental conditions critically modulate the association between growth and RTL. Previous work on the roe deer populations we studied demonstrates that overall age–telomere relationships sharply differ between them [65]. Our study extends this population-level observation by revealing the underlying individual correlates. In roe deer, heavier fawns at eight months of age had shortened telomeres only in the high-quality environment of Trois-Fontaines, whereas no such association was detectable in the harsher environment of Chizé. This seemingly counter-intuitive finding may reflect differences in realized growth potential. Evolutionary theory posits that organisms typically grow at sub-maximal, optimal rates to avoid the somatic costs associated with their physiological limits [2,3]. However, in a favourable environment like Trois-Fontaines, fawns may approach their physiological growth limits, thereby potentially incurring higher metabolic and cellular costs. Indeed, fawns at Trois-Fontaines were on average 3.27 ± 0.255 (s.e.) kg heavier at eight months of age compared with their Chizé counterparts, representing a massive approximately 25% difference in body mass. We posit that this elevated growth may push cellular machinery and metabolism towards their upper limits, thereby increasing oxidative damage and accelerating telomere attrition. By contrast, constrained growth in Chizé may prevent fawns from reaching the metabolic threshold at which telomere erosion becomes detectable. Our study provides empirical evidence that the association between early-life growth and TL in the wild could be modulated by environmental quality. Interestingly, the few previous studies that explicitly tested for environmental interactions reported opposite trends, with the negative associations of body size or growth observed primarily in lower-quality habitats (e.g. in juvenile salmon, *Salmo salar* [75], and a viviparous lizard, *Niveoscincus ocellatus* [76]). However, these studies focused on ectotherms, whose developmental dynamics, metabolic constraints and responses to environmental harshness fundamentally differ from those of large endotherms. To reach a heavy body mass in the favourable environment of Trois-Fontaines, roe deer have to sustain a rapid and continuous developmental trajectory. The physiological toll associated with this accelerated growth shares mechanistic similarities with the costs of compensatory growth. When individuals rapidly accumulate somatic tissue, such as king penguin chicks compensating for a growth delay [77] or laboratory rats undergoing diet-induced catch-up growth [78], the resulting surge in this accelerated growth is associated with an acceleration of telomere attrition. Our findings suggest that realizing a maximal developmental potential in a rich natural environment can be associated with similar physiological trade-offs in mammals in the wild.

Beyond the direct costs of early-life growth, other factors of variation we included in our models indicated that individual allocation strategies and environmental stringency both constrain somatic maintenance. In Soay sheep, individuals developing normal horns exhibited shorter telomeres compared with scurred or polled individuals, illustrating an additional cellular toll associated with the growth of secondary sexual traits. In roe deer, the impact of environmental quality was also reflected in broader telomere dynamics: RTL increased with age in the favourable habitat of Trois-Fontaines, but tended to decrease in the harsh environment of Chizé. Importantly, this apparent increase in RTL with age at Trois-Fontaines was not driven by the selective disappearance of poorer-quality individuals. Supplementary survival analyses revealed no significant association between RTL and mortality risk in this population (supplementary material, table S3), indicating that survival bias is highly unlikely to explain the observed patterns. Furthermore, a trend for shorter telomeres in males compared with females was only observed at Chizé, suggesting that the vulnerability of males in harsh populations [45,79] may extend to the cellular level. More specifically, this raises the possibility that environmental harshness (e.g. resource shortage) might exacerbate the physiological cost of growth, which could ultimately translate into shorter telomeres. This warrants further investigation in future studies.

In conclusion, our study on two large herbivores in the wild demonstrates that early-life telomere dynamics are highly dependent on both individual attributes, such as sex, and environmental context, such as environmental quality. Accounting for these sex- and population-specific effects is crucial, as they can otherwise obscure the underlying physiological trade-offs linking growth and TL in the wild.

## Supplementary Material



## Data Availability

Data are published in the supplementary material [80].

## Theme

One contribution of 21 to a theme issue ‘Developmental circuits in ageing’.
