# Supplementary material for: Sex and population mediate the ‘fast growth–short telomere’ association in two species of large herbivores monitored in the wild
Source: Philos Trans R Soc Lond B Biol Sci. 2026 Nov 12;381(1963):20250334. doi: 10.1098/rstb.2025.0334 (PMC13755916; doi:10.1098/rstb.2025.0334)
Supplement: Supplementary file 1 [file rstb.2025.0334.zip › rstb.2025.0334/rstb20250334_si_002.docx]

**Supplementary Information**

**Sex and population mediate the ‘fast growth - short telomere’ association in two species of large herbivores monitored in the wild**

**REMOT^1,2,†^ F., GAILLARD^2^ J.-M., REY^2^ B., GILOT-FROMONT^2,3^ E., PELLERIN^4^ M., DEBIAS^2^ F., GARCIA^2^ R., REGIS^2^ C., REVELLI^2^ P., SAUTIER^2^ E., DORRENS^5^ J., FROY^5^ H., SEEKER^5,6^ L.A., UNDERWOOD^5^ S.L., WILBOURN^5^ R.V., NUSSEY^5*^ D.H. & LEMAITRE^2*^ J.-F.**

**^1^** Université Côte d’Azur - CNRS - Inserm - Institute for Research on Cancer and Ageing, Nice (IRCAN), Medical School, Nice, France

^2^ Université de Lyon, Univ Lyon 1, CNRS, LBBE UMR 5558, F-69622 Villeurbanne, France

^3^ VetagroSup, Marcy l'Etoile, France

^4^ Office Français de la biodiversité, Direction de la Recherche et de l’Appui Scientifique, Service Conservation et Gestion Durable des Espèces Exploitées, Chateauvillain, 52210, France

^5^ Institute of Ecology & Evolution, School of Biological Sciences, University of Edinburgh, Edinburgh, UK

^6^ School of Bioengineering, Stanford University, USA

**^†^** Corresponding author: [florentin.remot@gmail.com](mailto:florentin.remot@gmail.com)

***** Both authors contributed equally to this work

**Supplementary Methods**

**(a) Survival analysis in Trois-Fontaine**

To investigate whether relative telomere length (RTL) influenced mortality risk in the Trois-Fontaines population, we performed a survival analysis using Cox proportional hazards model. THis analysis was conducted on a subset of 91 individuals for which reliable survival data (age last seen) was available. Individual sex was included as a covariate in the model to account for potential sex-specific survival probabilities. Individuals still alive at the end of the monitoring period were right-censored. The analysis was conducted using the *survival* package (version 3.8-3, [[1]](https://www.zotero.org/google-docs/?x1mg3R))

**Supplementary Results**

**Table S1: Studies of the relationship between post-natal growth parameters and telomere length and/or loss in wild vertebrates. d, day; PCA, principal component analysis; cort, corticosterone; RBC, red blood cell; WBC, white blood cell; RTL, relative telomere length; Exp, experimental studies (involving the manipulation of growth); Corr, correlative/observational studies; Exp/corr, studies that possessed an experimental design but where the treatment did not result in a significant modification of growth rates; TRFI, telomere restriction fragment with in-gel hybridization; TRFS, telomere restriction fragment followed by a Southern-Blot; ns, not significant; SVL, snout-vent length. Updated from Monaghan & Ozanne (2018). Bolded lines represent studies that have been published since 2018.**

**Table S2: Summary of the global model testing the predictors of relative telomere length (RTL) in roe deer fawns. Estimates, standard errors (SE) and P-value from the global model testing the effects of standardized birth mass and standardized juvenile mass on RTL in the subset of fawns from Trois-Fontaines. The reference level for sex is ‘female’. Statistically significant effects are in bold.**

| Traits | Estimate | SE | P-value |
| --- | --- | --- | --- |
| **Age at sampling** | **0.022** | **0.007** | **0.001** |
| Standardized body mass at 4 days of age | -0.077 | 0.062 | 0.220 |
| Standardized fawn mass (at 8 months of age) | -0.016 | 0.011 | 0.133 |
| Sex | 0.014 | 0.046 | 0.760 |

**(a) Survival analysis in Trois-Fontaine**

The survival analysis revealed no significant association between RTL and mortality risk in the Trois-Fontaines population (HR = 0.83, p-value = 0.89) (See **Table S3).** This indicates that individuals with shorter telomeres did not suffer from a higher mortality rate compared to individuals with longer telomeres. Consequently, the selective disappearance of poorer-quality individuals cannot explain the apparent increase in RTL with age observed in this population.

**Table S3: Summary of the Cox proportional hazards model testing the effect of relative telomere length (RTL) on survival in roe deer. Hazard ratios (HR), standard errors (SE) and P-values are extracted from the model testing the effects of RTL and sex on mortality risk in the subset of 91 individuals from Trois-Fontaines. The reference level for sex is ‘female’. Statistically significant effects are in bold.**

| Traits | HR | SE | P-value |
| --- | --- | --- | --- |
| RTL | 0.830 | 1.335 | 0.889 |
| Sex | 0.652 | 0.546 | 0.432 |

**References:**

[1.](https://www.zotero.org/google-docs/?86LKoR)	[Therneau TM. 2024 survival: A Package for Survival Analysis in R.](https://www.zotero.org/google-docs/?86LKoR)
